# Real-world effectiveness of osteoporosis treatments in Germany

**DOI:** 10.1007/s11657-022-01156-z

**Published:** 2022-08-31

**Authors:** James O’Kelly, Robert Bartsch, Nils Kossack, Julia Borchert, Marc Pignot, Peyman Hadji

**Affiliations:** 1 Amgen Ltd, Uxbridge, UK; 2 Amgen, Munich, Germany; 3 WIG2 GmbH, Leipzig, Germany; 4 Kantar GmbH, Health Division, Munich, Germany; 5 Frankfurt Center of Bone Health, Frankfurt, Germany; 6 Philipps-University of Marburg, Marburg, Germany

**Keywords:** Fracture rate, Germany, Postmenopausal osteoporosis, Real-world, Retrospective cohort

## Abstract

***Summary*:**

This observational study assessed the impact on the fracture incidence of osteoporosis medications in postmenopausal women in Germany. Continued treatment with osteoporosis medications was associated with reductions of fracture rates in a real-world setting.

**Purpose:**

The efficacy of osteoporosis medications has been demonstrated in clinical trials, but a lack of evidence exists of their real-world effectiveness. This real-world study assessed the treatment patterns and impact on the fracture incidence of osteoporosis medications in postmenopausal women in Germany.

**Methods:**

This cohort study used data from the WIG2 benchmark database, a German anonymised healthcare claims database. All women ≥ 50 years of age with ≥ 1 prescription for osteoporosis medication between 1 January 2013 and 31 December 2017 were included. The primary outcome was treatment effectiveness, evaluated as the change in fracture incidence after initiating treatment. Fracture types included all fractures, clinical vertebral, hip and wrist/forearm. Fracture incidence was assessed during the early-treatment period (0–3 months) and the on-treatment period (4–12, 13–24, 25–36 and 37–48 months).

**Results:**

Baseline covariates and treatment patterns were determined for 41,861 patients. The median duration of therapy was longer with denosumab (587 days) than with intravenous ibandronate (451 days), intravenous zoledronate (389 days) or oral bisphosphonates (258 days). The baseline incidence rate of all fractures was higher in patients receiving denosumab than in those receiving other treatments (87.6, 78.2, 56.6 and 66.0 per 1000 person-years for denosumab, oral bisphosphonates, intravenous ibandronate and intravenous zoledronate, respectively). Rates of all fractures declined with continued denosumab (by 38%, 50%, 56% and 67% at 12, 24, 36 and 48 months, respectively) and oral bisphosphonates (by 39%, 44%, 49% and 42%, respectively) treatment.

**Conclusion:**

Continued treatment with osteoporosis medications was associated with reductions of fracture rates in a real-world setting.

**Supplementary Information:**

The online version contains supplementary material available at 10.1007/s11657-022-01156-z.

## Introduction

Osteoporosis is the most common bone disease in humans and is characterised by low bone mass and deterioration of bone tissue. It can lead to compromised bone strength and an increase in the risk of fractures [1]. It is estimated that approximately 25.5 million women and 6.5 million men in the European Union (EU) have osteoporosis [2]. The annual number of osteoporotic fractures in the EU is expected to rise from 4.3 million in 2019 to 5.3 million in 2034 [2]. The incidence of fractures and type of fracture vary by geographic region [2, 3]. In the EU, Germany has the highest number of fractures, approximately 800,000 incident fractures in total, owing to the large population size and comparatively high fracture rate [2]. This figure is predicted to increase over time because of the ageing population [4].

It is well established that osteoporosis is associated with a poor health-related quality of life [5]. Osteoporosis-related fractures not only have an enormous impact on patients’ health-related quality of life but also on healthcare systems. The cost of osteoporosis in the EU has been estimated at approximately €37 billion.

Current treatments for osteoporosis aim to inhibit bone resorption and/or stimulate bone formation [6]. Anti-resorptive therapies, such as bisphosphonates or denosumab, are used to increase bone strength in individuals with osteoporosis, and they are the most commonly used therapies in the treatment of osteoporosis [7]. According to the German osteoporosis guidelines, the recommended medications for fracture risk reduction in postmenopausal women are anti-resorptive agents (alendronate, denosumab, ibandronate, risedronate or zoledronate), hormone replacement therapy (HRT; oestrogen and progesterone), parathyroid hormones (teriparatide) and selective oestrogen receptor modulators (bazedoxifene or raloxifene) [8]. The efficacies of current treatments for osteoporosis have been demonstrated in clinical trials [9–12], but there is a lack of evidence of their real-world effectiveness in the German population [8].

Real-world studies are critical in providing evidence of treatment effectiveness in clinical practice [13]. Indeed, although randomised clinical trials (RCTs) provide robust evidence in evaluating the safety and efficacy of new therapeutic agents, limiting inclusion and exclusion criteria mean that trial populations are often not representative of the patient populations found in clinical practice [14]. The patient populations in real-world studies are more representative of clinical practice than those selected in RCTs. Furthermore, real-world studies can have a larger sample size and assess treatment patterns across a much broader range of outcomes [14, 15].

The aim of this cohort study was to assess the treatment patterns and impact of osteoporosis medications on fracture incidence in postmenopausal women in routine care in Germany. Treatment effectiveness was assessed within individual treatment cohorts, by examining longitudinal changes in the fracture incidence from the initiation of treatment.

## Methods

### Population and data source

This retrospective cohort study used pre-existing claims data from the Scientific Institute for Health Economics and Health Systems Research (Wissenschaftliches Institut für Gesundheitsökonomie und Gesundheitssystemforschung [WIG2]) benchmark database, a German anonymised healthcare claims database with longitudinal data on approximately 4.5 million Germans. The dataset, shown to be representative of the German statutory health insurance population with regard to age, sex and morbidity, [16] covered a time period limited to 2011–2018, with claim codes and algorithms for defining outcomes and covariates based on published studies with agreement between all collaborating study parties (Amgen, Kantar, WIG2).

Patients were eligible for inclusion if they were women 50 years of age or older (consistent with previously published clinical data [17, 18] and represents the mean age of menopause of German women. In addition, postmenopausal women or women over the age of 50 have the greatest risk of developing osteoporosis [19]) and had received a prescription for at least one of the following osteoporosis medications between 1 January 2013 and 31 December 2017: denosumab; intravenous bisphosphonates (ibandronate or zoledronate); oral bisphosphonates; teriparatide; raloxifene; or HRT (a combination of progesterone and oestrogen, or oestrogens alone for women who had undergone a hysterectomy). Patients were excluded if they had less than 24 months of available medical history, a diagnosis of another disease that had a possible influence on bone health (e.g. active cancer of any kind, Paget’s disease or osteogenesis imperfecta), or had received a prescription of the first osteoporosis medication used (i.e. index treatment) in the past 24 months (pre-index treatment). Patients were permitted to have received treatments other than the index treatment in the past 24 months but were not permitted to have received a prescription of the index treatment in the past 24 months. To reduce selection bias, the selection of patients was not based on a sampling method, and the entire cohort was observed, regardless of treatment, health status (except for patients with cancer or Paget’s disease) or other considerations.

A minimum follow-up period of 6 months was required to analyse the fracture risk. Patients were assigned to only one treatment group, according to the order used above, based on a hierarchical approach published previously [20]. The index date was defined as the first use of the index treatment between 1 January 2013 and 31 December 2017. The follow-up period started on the index date and ended on the first of the following events: discontinuation of or switch from the index treatment; patient death; end of insurance; or calendar date 31 December 2018.

### Outcomes

The primary outcome of the study was the evaluation of the change in fracture risk after treatment initiation for postmenopausal women receiving osteoporosis treatment. The analysis was carried out for the whole population and was stratified by the type of treatment and type of fracture. Fractures were defined by the occurrence during the follow-up period, including the date of either: one inpatient diagnosis; or one outpatient diagnosis in combination with a fracture-related inpatient or outpatient procedure. Fracture types included the following osteoporosis-related fractures: all fractures (hip, clinical vertebral, wrist/forearm, humerus, clavicle, pelvis, femur) or according to selected fracture types: clinical vertebral; hip; or wrist/forearm (Supplementary Table 1). Patients may experience multiple fractures over the study period, and all fractures were considered in the analysis of fracture outcomes. Fracture diagnoses occurring within 3 months of each other and associated with fractures of the same body part were considered the same fracture event.

Treatment patterns were estimated, including treatment duration and switch from index treatment. Treatment duration was measured as continuous treatment with the index treatment until a gap of more than 60 days, without either filling a new prescription after the expected refill date or a switch to another treatment. Treatment switching was defined as starting a new therapy within the 60-day window.

### Fracture incidence rates’ assessment

**Fig. 1:**
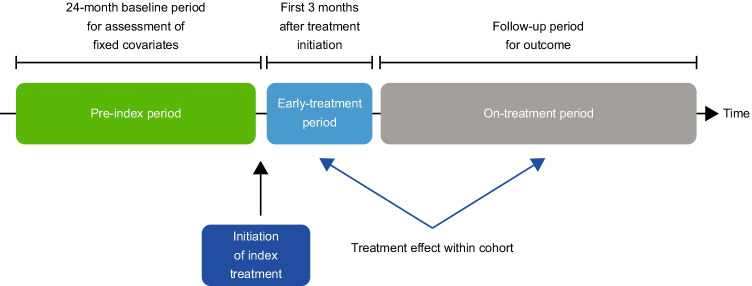
Schematic showing the pre-index, early-treatment and on-treatment periods

The fracture incidence rate (per 1000 person-years) was assessed during the following periods consistent with previously described real-world effectiveness studies [17, 20] (Fig. 1): (1) the initial 3-month period after starting therapy (which was chosen to represent the baseline fracture risk used in previous longitudinal studies of real-world effectiveness [17, 20] which is based on observations from clinical studies of bisphosphonates and denosumab which show no clear effect on fracture outcomes versus placebo for at least the first 6 months [21, 22]) and (2) the on-treatment period, which was defined as the subsequent time on-treatment after the first 3 months post-index until the end of follow-up.

Fracture incidence rates were assessed in the early-treatment period to establish the baseline fracture risk, and treatment effectiveness was evaluated as the change in fracture incidence rates from the early-treatment period using an own-control analysis [20]. Fracture incidence rates were compared between the early-treatment and on-treatment periods using incidence rate ratios (IRRs). The on-treatment period was assessed for the periods 4–12, 13–24, 25–36 and 37–48 months after index.

The change in fracture risk was assessed using an as-treated analysis approach, in which patients were followed from the index date until the earliest of either treatment discontinuation, a switch to a different treatment group, patient death or calendar date 31 December 2018.

### Statistical analysis

Descriptive statistics were calculated for all study variables. For categorical variables, the absolute number of patients and the proportion of patients relative to the total sample size in each treatment group were reported. For each treatment group, IRRs with 95% confidence intervals (CIs) were reported. A post hoc trend analysis was conducted using a weighted linear model to assess the change in fracture rates over time, during on-treatment periods. A conditional Poisson regression analysis was conducted to explore the impact of age during periods of longer follow-up in an elderly population. The regression coefficient estimated the difference in the log of the expected fracture counts over the whole study period for a given predictor variable (i.e. an additional year of age given while other variables remain constant). IRRs were estimated to compare fracture incidence rates with adjustment for age as a potential time-varying confounder. Missing data were not imputed.

## Results

### Patient characteristics

**Fig. 2:**
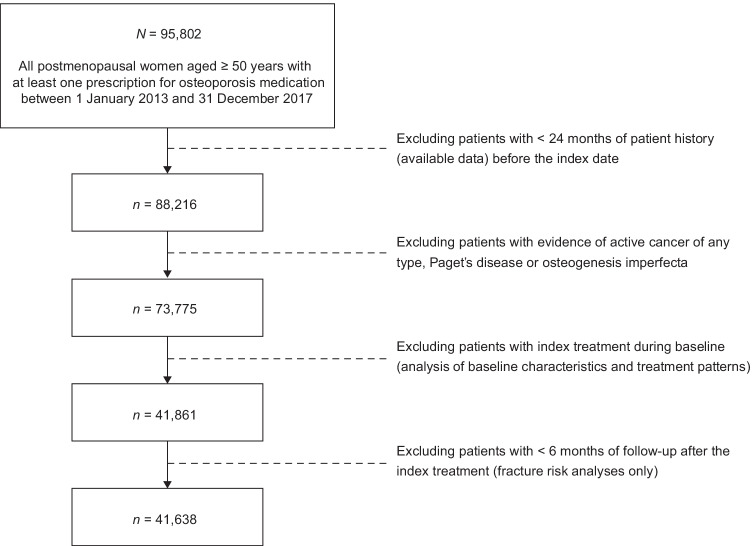
Patient flow diagram

In total, 95,802 women 50 years of age or older who received an osteoporosis medication were identified (Fig. 2). Patients with less than 24 months of available baseline data prior to the index date were excluded, reducing the sample size to 88,216. Excluding patients with evidence of active cancer of any kind, Paget's disease or osteogenesis imperfecta further reduced the number of patients to 73,775. Only newly treated patients who met the above criteria and had not received a prior prescription of the index treatment in the baseline period were included. Baseline covariates and treatment patterns were determined for 41,861 patients who met the above criteria. In total, 41,638 patients had a minimum follow-up period of 6 months for analysis of fracture risk.

Patient characteristics were reported for all therapies (Table 1). Mean age was similar among patients receiving anti-resorptive therapies, including denosumab, oral bisphosphonates, intravenous ibandronate and intravenous zoledronate. Of note, patients who were receiving HRT were younger than those in the other treatment groups (Table 1). The Charlson comorbidity index weight was highest in the teriparatide group (2.84) followed by the denosumab group (2.24) and was lowest in those receiving HRT (0.87).

**Table 1:** Baseline patient demographics and clinical characteristics

Demographic or characteristic	Denosumab (*N* = 3495)	Oral bisphosphonates (*N* = 13,134)	Intravenous ibandronate (*N* = 1801)	Intravenous zoledronate (*N* = 379)	Teriparatide (*N* = 43)	Raloxifene (*N* = 120)	HRT (*N* = 22,889)
Age, years, mean	72.8	71.9	71.9	69.5	72.2	68.0	56.4
Age group, years, *n* (%)							
50–59	297 (8)	1591 (12)	191 (11)	60 (16)	8 (19)	32 (27)	17,975 (79)
60–69	848 (24)	3265 (25)	459 (25)	123 (32)	4 (9)	33 (28)	3152 (14)
70–79	1520 (43)	5477 (42)	797 (44)	145 (38)	23 (53)	37 (31)	1468 (6)
≥ 80	830 (24)	2801 (21)	354 (20)	51 (13)	8 (19)	18 (15)	294 (1)
Mean CCI weight	2.24	2.06	2.03	1.92	2.84	1.22	0.87
Comorbidities^a^, *n* (%)							
Congestive heart failure	655 (19)	2401 (18)	284 (16)	60 (16)	17 (40)	10 (8)	896 (4)
COPD	478 (14)	1867 (14)	236 (13)	41 (11)	10 (23)	6 (5)	1245 (5)
Depression	1176 (34)	4166 (32)	645 (36)	136 (36)	22 (51)	23 (19)	7192 (31)
Epilepsy	507 (15)	1594 (12)	236 (13)	55 (15)	8 (19)	13 (11)	1415 (6)
Hyperlipidaemia	1802 (52)	6696 (51)	908 (50)	166 (44)	20 (47)	50 (42)	6278 (27)
Moderate or severe renal disease	683 (20)	1714 (13)	230 (13)	32 (8)	10 (23)	11 (9)	621 (3)
Renal insufficiency	565 (16)	1538 (12)	209 (12)	26 (7)	8 (19)	7 (6)	512 (2)
Rheumatoid arthritis	476 (14)	1284 (10)	209 (12)	64 (17)	5 (12)	7 (6)	788 (3)
Senility	240 (7)	736 (6)	113 (6)	15 (4)	6 (14)	5 (4)	115 (1)
Type 1 or 2 diabetes	565 (16)	2429 (18)	307 (17)	58 (15)	8 (19)	15 (13)	1866 (8)
Prior medication, *n* (%)							
Proton pump inhibitors	2147 (61)	7263 (55)	1158 (64)	244 (64)	36 (84)	53 (44)	8788 (38)
Systemic glucocorticoids	648 (19)	2279 (17)	352 (20)	90 (24)	11 (26)	8 (7)	1627 (7)
Osteoporosis history, *n* (%)							
BMD test	924 (26)	2051 (16)	411 (23)	79 (21)	10 (23)	22 (18)	187 (1)
Prior osteoporosis treatment	1901 (54)	587 (4)	872 (48)	180 (47)	4 (9)	12 (10)	1 198 (5)
Prior fracture, *n* (%)							
Any	574 (16)	2284 (17)	261 (14)	48 (13)	24 (56)	8 (7)	178 (1)
Clinical vertebral	307 (9)	1155 (9)	127 (7)	30 (8)	17 (40)	4 (3)	28 (< 1)
Hip	105 (3)	555 (4)	56 (3)	8 (2)	5 (12)	2 (2)	11 (< 1)
Wrist/forearm	98 (3)	356 (3)	44 (2)	6 (2)	2 (5)	1 (1)	59 (< 1)

^a^Comorbidities included are based on risk factors listed in the DVO guidelines. Comorbidities present in less than 5% of patients in all treatment cohorts were not listed: chronic inflammatory bowel disease, monoclonal gammopathy, nutritional deficiencies or eating disorders, and Parkinson’s disease

BMD, bone mineral density; CCI, Charlson comorbidity index; COPD, chronic obstructive pulmonary disease; DVO, Dachverband Osteologie; HRT, hormone replacement therapy

Patients receiving teriparatide as their index treatment had higher rates of baseline comorbidities than those in other treatment groups (Table 1). Patients receiving anti-resorptive agents had similar levels of comorbidities with the exception of moderate or severe renal disease, which was highest in the denosumab group (20%, 13%, 13% and 8% for denosumab, oral bisphosphonates, intravenous ibandronate and intravenous zoledronate, respectively).

Previous use of osteoporosis treatment was highest in patients who received denosumab (54%) and lowest in those who received oral bisphosphonates (4%). A prior fracture had been experienced by similar proportions of patients who received denosumab (16%) and intravenous or oral bisphosphonates (13–17%) and was much higher in those who received teriparatide (56%) (Table 1).

### Treatment patterns

Treatment patterns during follow-up are described in Table 2 and Supplementary Fig. 1. The median duration of therapy was longer with denosumab (587 days) than with intravenous ibandronate (451 days), intravenous zoledronate (389 days) or oral bisphosphonates (258 days) (Table 2). Patients receiving denosumab who had previously received osteoporosis therapy had a longer median duration of treatment (614 days) than those with no prior therapy (560 days). This pattern was repeated for the intravenous bisphosphonates but reversed for oral bisphosphonates. Treatment switching was infrequent. In each treatment group, fewer than 3% of patients switched from their index treatment during the study period, and most patients discontinued the index treatment without switching.

**Table 2:** Treatment durations

	**Denosumab (*N* = 3495)**	**Oral bisphosphonates (*N* = 13,134)**	**Intravenous ibandronate (*N* = 1801)**	**Intravenous zoledronate (*N* = 379)**	**Teriparatide** **(*N* = 43)**	**Raloxifene (*N* = 120)**	**HRT (*N* = 22,889)**
Median (IQR) treatment duration (days)^a^							
Overall	587 (701)	258 (638)	451 (683)	389 (457)	442 (559)	160 (591)	149 (269)
No prior treatment	560 (817)	259 (637)	372 (689)	358 (399)	473 (559)	160 (553)	149 (265)
Prior treatment	614 (709)	234 (647)	501 (727)	559 (696)	356 (385)	84 (732)	148 (341)

^a^Treatment duration was measured as continuous treatment with index therapy until a gap of more than 60 days without filling a new prescription after the expected refill date or treatment switching occurred

HRT, hormone replacement therapy; IQR, interquartile range

### Fracture incidences

**Fig. 3:**
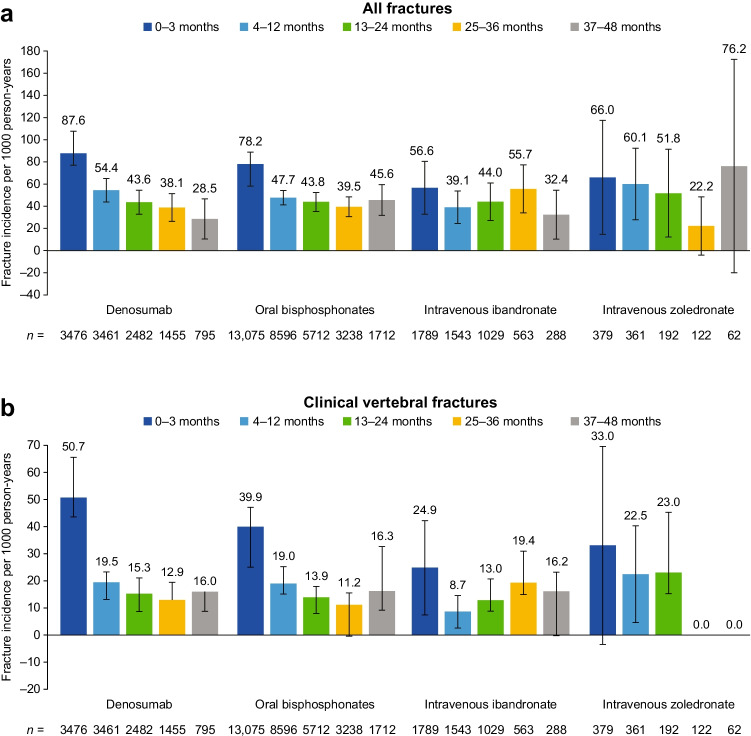

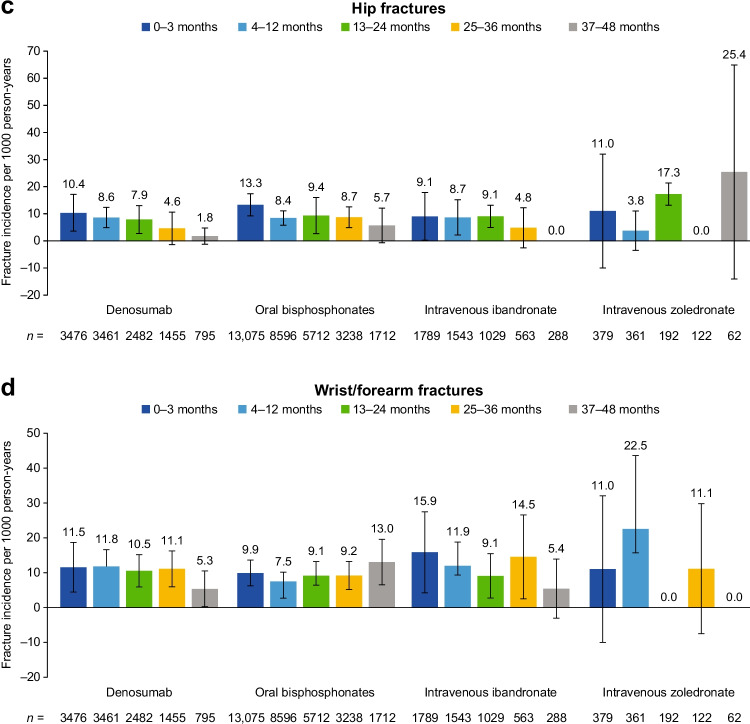
Fracture incidence rate during the early-treatment period (0–3 months) and on-treatment periods (4–12, 13–24, 25–36 and 37–48 months) in patients receiving anti-resorptive therapy

Figure 3 shows fracture incidences during different periods following initiation of treatment, and Table 3 shows IRRs comparing the change in the fracture rate from the early-treatment period to subsequent on-treatment periods. The incidence rate of all fractures during the early-treatment period was highest in patients receiving denosumab as compared with those receiving other anti-resorptive therapies (87.6, 78.2, 56.6 and 66.0 for denosumab, oral bisphosphonates, intravenous ibandronate and intravenous zoledronate, respectively) (Fig. 3a). The incidence rate for clinical vertebral fractures in the early-treatment period was also highest in those receiving denosumab as compared with those receiving oral bisphosphonates, intravenous ibandronate or intravenous zoledronate (50.7, 39.9, 24.9 and 33.0, respectively) (Fig. 3b).

**Table 3:** IRRs comparing fracture incidence rates during the early-treatment period (0–3 months) with the on-treatment periods (4–12, 13–24, 25–36 and 37–48 months) in patients receiving anti-resorptive therapy

Time (months)	Denosumab		Oral bisphosphonates		Intravenous ibandronate		Intravenous zoledronate	
	***N***	**IRR (95% CI)**	***N***	**IRR (95% CI)**	***N***	**IRR (95% CI)**	***N***	**IRR (95% CI)**
**All fractures**								
4–12	120	0.62 (0.47–0.83)	249	0.61 (0.51–0.73)	36	0.69 (0.41–1.15)	16	0.91 (0.36–2.33)
13–24	83	0.50 (0.36–0.68)	192	0.56 (0.46–0.68)	34	0.78 (0.46–1.30)	9	0.79 (0.28–2.21)
25–36	42	0.44 (0.30–0.65)	95	0.51 (0.40–0.64)	23	0.98 (0.56–1.73)	2	0.34 (0.07–1.67)
37–48	18	0.33 (0.19–0.56)	56	0.58 (0.44–0.78)	6	0.57 (0.23–1.39)	3	1.15 (0.29–4.62)
**Clinical vertebral fractures**								
4–12	43	0.38 (0.25–0.59)	99	0.47 (0.36–0.62)	8	0.35 (0.14–0.87)	6	0.68 (0.17–2.73)
13–24	29	0.30 (0.19–0.48)	61	0.35 (0.26–0.48)	10	0.52 (0.22–1.22)	4	0.70 (0.16–3.12)
25–36	14	0.26 (0.14–0.47)	27	0.28 (0.19–0.43)	8	0.78 (0.31–1.93)	–	–
37–48	9	0.32 (0.15–0.65)	20	0.41 (0.25–0.66)	3	0.65 (0.18–2.33)	–	–
**Hip fractures**								
4–12	19	0.83 (0.38–1.84)	44	0.63 (0.41–0.97)	8	0.96 (0.29–3.18)	1	0.34 (0.02–5.46)
13–24	15	0.76 (0.33–1.74)	41	0.70 (0.45–1.09)	7	1.00 (0.29–3.42)	3	1.57 (0.16–15.09)
25–36	5	0.45 (0.15–1.33)	21	0.66 (0.39–1.12)	2	0.53 (0.10–2.92)	–	–
37–48	1	0.17 (0.02–1.36)	7	0.43 (0.19–0.96)	–	–	1	2.31 (0.14–36.93)
**Wrist/forearm fractures**								
4–12	26	1.02 (0.49–2.12)	39	0.75 (0.47–1.22)	11	0.75 (0.29–1.94)	6	2.05 (0.25–17.01)
13–24	20	0.91 (0.43–1.95)	40	0.92 (0.57–1.49)	7	0.57 (0.20–1.63)	–	–
25–36	12	0.96 (0.42–2.23)	22	0.93 (0.53–1.61)	6	0.92 (0.31–2.73)	1	1.01 (0.06–16.14)
37–48	3	0.46 (0.13–1.68)	16	1.32 (0.71–2.42)	1	0.34 (0.04–2.77)	–	–

CI, confidence interval; IRR, incidence rate ratio; *N*, number of fractures

A reduction in the incidence rate for all fractures was observed during treatment with all medications, with the exception of intravenous zoledronate at 37–48 months (Fig. 3a). In the denosumab cohort, rates of all fractures declined from the early-treatment period (0–3 months after treatment initiation) to the first on-treatment period (4–12 months after treatment initiation) and continued to decline in the subsequent 12-month on-treatment periods (decreases from the early-treatment period of 38%, 50%, 56% and 67% at 12, 24, 36 and 48 months, respectively).

The rates for all fractures in the oral bisphosphonates cohort were lower during all on-treatment periods than the early-treatment period (decreased by 39%, 44%, 49% and 42% at 12, 24, 36 and 48 months, respectively). The incidence rates of all fractures fluctuated in the intravenous ibandronate cohort, declining during months 4–12, then increasing during months 25–36, and declining again during months 37–48 (decreases from the early-treatment period of 31%, 22%, 2% and 43%, respectively).

The pattern of clinical vertebral fracture rates was similar to that observed for all fractures. Denosumab patients had the highest rate of clinical vertebral fractures during the early-treatment period, and the incidence rates were lower during on-treatment periods in all treatment cohorts (decreased by 62–74%). Fracture incidence rates were also lower in the oral bisphosphonate cohort (decreased by 53–72%) and the intravenous ibandronate cohort (decreased by 22–65%).

The incidence rates of hip fractures in all cohorts were lower than the incidence rates of clinical vertebral fractures. In the denosumab cohort, hip fracture rates declined in each subsequent treatment period (decreases from the early-treatment period of 17%, 24%, 55% and 83% at 12, 24, 36 and 48 months, respectively). In the oral bisphosphonate cohort, hip fracture rates were also lower during on-treatment periods than the early-treatment period (decreases from the early-treatment period of 37%, 30%, 34% and 57% at 12, 24, 36 and 48 months, respectively).

The rate of wrist/forearm fractures did not appreciably change during 36 months of treatment in any of the cohorts.

Analysis of trends over time in the denosumab cohort showed a decrease in fracture rates per on-treatment period of − 8.36, − 2.01, − 2.15 and − 1.46 per 1000 person-years for all, clinical vertebral, hip and wrist/forearm fractures, respectively. The same analysis in the oral bisphosphonate cohort showed changes per on-treatment period of − 2.09, − 2.16, − 0.44 and + 1.46 per 1000 person-years.

Patient and/or event numbers were low in the intravenous zoledronate, teriparatide, raloxifene and progesterone and oestrogen combination cohorts, leading to wide 95% CIs; particularly at later time periods and for individual fracture types (Fig. 3 and Supplementary Fig. 2). Nonetheless, the incidence rate of all fractures in the intravenous zoledronate cohort appeared to decline during the first 36 months of treatment.

### Stratified analysis

In analyses stratified by prior fracture, the incidence of all fractures during the early-treatment period was 1.8- to 2.7-fold higher among patients with a prior fracture than those with no history of a fracture (Supplementary Fig. 3). Consistent with the overall analysis, the incidence rate of all fractures declined during continued treatment with denosumab, oral bisphosphonates or intravenous ibandronate, in patients with or without prior fracture. In analyses stratified by prior osteoporosis treatment, incidence rates of all fractures during the early-treatment period were 1.4–3.8 times higher among patients with no prior osteoporosis treatment than those who had a prior treatment (Supplementary Fig. 4). Fracture incidence rates during treatment declined in both groups, and successive reductions in the fracture rate were observed with continued denosumab treatment regardless of treatment history.

### Sensitivity analysis

A Poisson regression analysis showed that per year of ageing, the risk of a fracture increased by 5% across all fractures (IRR = 1.05, 95% CI: 1.04–1.05). Analysis of specific types of fractures showed that the risk of fracture increased per year of ageing by 2–8%.

## Discussion

The present study assessed the characteristics of postmenopausal women receiving treatment for osteoporosis in Germany and the impact of treatment on the occurrence of osteoporosis-related fractures. These data demonstrate that all standard therapies for osteoporosis reduced the fracture incidence rate during treatment. Notably, during treatment with denosumab, the incidence rates of all fractures and hip fracture continued to decline for up to 48 months of treatment. The baseline fracture risk appeared lowest in patients receiving HRT or raloxifene followed by those receiving oral and intravenous bisphosphonates or denosumab and highest in those receiving teriparatide. The HRT and raloxifene cohorts were younger in age than other treatment cohorts and had fewer comorbidities, less bone mineral density (BMD) testing and fewer prior fractures, indicating a predominantly primary prevention strategy in these patients.

The treatment duration was longer with denosumab than with other anti-resorptive therapies. These results are consistent with the findings of a previous study, which demonstrated that persistence with denosumab was 1.5–2 times higher than with intravenous or oral bisphosphonates [23]. In our study, patients receiving denosumab who had previously received osteoporosis therapy also had longer median duration of treatment than those with no prior therapy. However, patients receiving oral bisphosphonates who had previously received osteoporosis therapy had a shorter median duration of treatment than those with no prior therapy. These results are consistent with the findings of the study by Freemantle et al., which showed that patients reported greater satisfaction with denosumab and preferred it over oral alendronate [24].

The efficacy of osteoporosis medications has been demonstrated in RCTs [21, 25–27]. In a large meta-analysis of RCTs evaluating the risk of hip, vertebral or non-vertebral fractures in postmenopausal women, a significant reduction in fracture risk was observed with osteoporosis therapies [26]. In a recent meta-regression analysis of 69 RCTs, it was demonstrated that anti-resorptive treatments were beneficial for fracture risk reduction among postmenopausal women [27]. Furthermore, in the FREEDOM study, a large randomised study of denosumab in postmenopausal women with osteoporosis, denosumab reduced the risk of vertebral fractures by 68% compared with placebo in addition to demonstrating significant reductions in non-vertebral and hip fracture risk [21].

Although clinical trial data have demonstrated that anti-resorptive agents can reduce the risk of fractures, it is important to understand whether osteoporosis medications reduce fracture rates in the real-world population of postmenopausal women with osteoporosis. A study using an own-control analysis of Swedish registry data showed that fracture incidence declined after treatment with oral or intravenous bisphosphonates [17]. When the same method was applied to the US Medicare database, it was found that the fracture incidence similarly declined after treatment with denosumab, zoledronic acid, teriparatide or oral bisphosphonates [20]. Such treatment effectiveness data have not been reported in Germany, where patient risk factors and treatment patterns may differ from those previously reported in other countries [3]. Indeed, to our knowledge, this is the first time this particular dataset of claims data has been examined to assess fracture rates in Germany. Furthermore, in the current analysis we have examined the fracture incidence during different follow-up periods, allowing an evaluation of the change in fracture rate over time.

The current study uses a previously described real-world effectiveness method: a within-cohort analysis that can determine the change in fracture risk after treatment initiation [17, 20]. Using this approach, patients act as their own controls and are followed from treatment initiation through follow-up, which accounts for confounders that remain stable over time. In this study, the first 3 months after treatment initiation were chosen to represent the baseline fracture risk: prior clinical studies have shown little difference in the incidence of fracture outcomes between denosumab and placebo for at least 6 months after treatment initiation [21]. Furthermore, recent studies have used fractures occurring in the first 3 months as ‘negative control outcomes’ to detect residual confounding on the assumption that there is no measurable treatment effect on fracture within the first 3 months and that this, therefore, represents a measure of baseline fracture risk [28]. In this real-world study, using this within-cohort approach, these data show that continued treatment with osteoporosis medications is associated with reductions of fracture rates in clinical practice. The incidence rate of all fractures and clinical vertebral fractures during the early-treatment period was highest in those receiving denosumab, as compared with those receiving other anti-resorptive therapies, indicating that the baseline fracture risk at treatment initiation was highest in the denosumab group. Ongoing treatment with denosumab led to continued decreases in the incidence rates of these fractures, as well as of hip fractures; this pattern of continued decline in fracture risk was not observed in other treatment groups. These observations are consistent with results from clinical studies: longer-term treatment with denosumab (up to 10 years in the FREEDOM extension study) was associated with continued increases in BMD and additional reductions in non-vertebral fractures [29, 30]. In contrast, clinical studies of bisphosphonates showed no additional fracture reductions with prolonged treatment [31–33].

In recent years, the choice of therapies available for the treatment of postmenopausal osteoporosis has increased significantly [34]. However, despite the availability of a wide range of effective therapies, a large proportion of women with osteoporosis in Germany are not receiving treatment [35]. This treatment gap is as high as 90% among women older than 70 years old of age who are at increased risk of fracture, 76% in those eligible for treatment [2] and 60% among those with an osteoporotic fracture [36]. The treatment gap has increased across Europe in the past 10 years and remained largely unchanged in Germany [2]. The causes of the continued treatment gap are unclear, but strategies to improve the provision of treatment to individuals at high risk of fracture will include increasing awareness among patients and physicians of the burden of osteoporosis, targeted risk assessment, and prioritisation of osteoporosis in national policy [2, 37].

A limitation of this study in the analysis of fracture incidence rates is the small numbers of patients and fracture events in some treatment cohorts and stratification groups. This was apparent in the intravenous zoledronate cohort, which included only six events of any fracture in the early-treatment period, and led to variation in point estimates for fracture over time and wide CIs in estimates of fracture incidences and IRRs. In general, this study is susceptible to the limitations associated with observational studies using administrative claims data. Fracture outcomes were determined using algorithms previously validated or used in other studies; although this approach may not eliminate misclassification, we do not anticipate any differential misclassification based on treatment. Another limitation concerns the use of the within-cohort approach in which patients act as their own controls. Although this method accounts for confounders that remain stable over time, it does not account for risk variables that may change over time including differential censoring between cohorts, weight/body mass index and comorbidities. Fracture rate data for treatments not in the anti-resorptive class are included in the supplemental material for completeness, but conclusions cannot be drawn on effectiveness owing to the small event numbers. There were differences in fracture risks between treatment groups (as demonstrated by differences in the fracture incidence rate during the early-treatment period), and no adjustments were made for differences in fracture risks between groups; therefore, direct comparisons of relative treatment effects between therapies should be avoided. Given that the risk of fractures within the treatment cohort was assessed in successive periods, distortions may occur owing to the increased age and the associated increase in fracture risk. However, the maximum on-treatment follow-up period did not exceed 48 months, so the age-related increase in fracture risk is expected to be minimal. A Poisson regression analysis found that the incidence of any fracture increased by 5% per additional year of age at index. Nonetheless, the fracture incidence rate during the on-treatment periods generally remained below that during the early-treatment period in the denosumab, oral bisphosphonates and intravenous ibandronate cohorts. A post-treatment analysis was beyond the scope of this study; however, it would be of interest in future studies to examine the fracture rates after treatment discontinuation.

In conclusion, in this real-world study, continued treatment with osteoporosis medications was associated with reductions of fracture rates. Continued reductions in fracture incidence were observed with denosumab treatment for up to 4 years. Differences in early-treatment period fracture rates indicate that patients’ individual fracture risk should be considered when selecting treatment options.

## Supplementary Information

Below is the link to the electronic supplementary material.

Supplementary file1 (DOC 88 kb)

Supplementary file2 (DOCX 15 kb)

Supplementary file3 (PDF 409 kb)

Supplementary file4 (PDF 439 kb)

Supplementary file5 (PDF 430 kb)

Supplementary file6 (PDF 431 kb)

Supplementary file7 (PDF 432 kb)

Supplementary file8 (PDF 431 kb)

## Data Availability

Qualified researchers may request data from Amgen clinical studies. Complete details are available at https://wwwext.amgen.com/science/clinical-trials/clinical-data-transparency-practices.

## Code availability

n/a.
