# Supplementary material for: Real-world effectiveness of osteoporosis treatments in Germany
Source: Arch Osteoporos. 2022 Aug 31;17(1):119. doi: 10.1007/s11657-022-01156-z (PMC9512727; doi:10.1007/s11657-022-01156-z)
Supplement: Supplementary file 2 — Supplementary file2 (DOCX 15 kb) [file 11657_2022_1156_MOESM2_ESM.docx]

**Supplementary material**

**Supplementary Table 1.** Fracture codes

| Hip | S72.0, S72.1, S72.2  M80.05, M84.45 |
| --- | --- |
| Clinical Vertebral | S12.0, S22.0, S22.1 S32.0  M48.50, M80.08, M84.48 |
| Wrist/forearm | S62.0 / S52.X  M80.03, M80.83 |
| Humerus | S42.2, S42.3, S42.4  M80.02 |
| Clavicle | S42.0 |
| Pelvis | S32.3, S32.4, S32.5, S32.8 |
| Leg (femur) | S72.3, S72.4, S72.8, S72.9, S79.1 |

**Supplementary Fig. 1** Proportions of patients receiving treatment during the early-treatment period (0–3 months) and on-treatment periods (4–12, 13–24, 25–36 and 37–48 months)

HRT, hormone replacement therapy

**Supplementary Fig. 2** Fracture incidence rate during the early-treatment period (0–3 months) and on-treatment periods (4–12, 13–24, 25–36 and 37–48 months) in patients receiving teriparatide^a^, raloxifene, a combination of progesterone and oestrogen or oestrogen for women who had undergone a hysterectomy.

Errors bars display 95% confidence intervals

Teriparatide data are shown to month 24,because this is the maximum permitted treatment duration per the summary of product characteristics and was the maximum observable treatment duration presented in Supplementary Fig 1

**Supplementary Fig. 3** Incidence rate of all fractures during the early-treatment (0–3 months) and on-treatment (4–12, 13–24, 25–36 and 37–48 months) periods in patients receiving anti-resorptive therapy, by prior fracture

Errors bars display 95% confidence intervals

**Supplementary Fig. 4** Incidence rate of all fractures during the early-treatment (0–3 months) and on-treatment (4–12, 13–24, 25–36 and 37–48 months) periods in patients receiving anti-resorptive therapy, by prior treatment history

Errors bars display 95% confidence intervals
