# Supplementary figures and images for: Real-world effectiveness of osteoporosis treatments in Germany
Source: Arch Osteoporos. 2022 Aug 31;17(1):119. doi: 10.1007/s11657-022-01156-z (PMC9512727; doi:10.1007/s11657-022-01156-z)

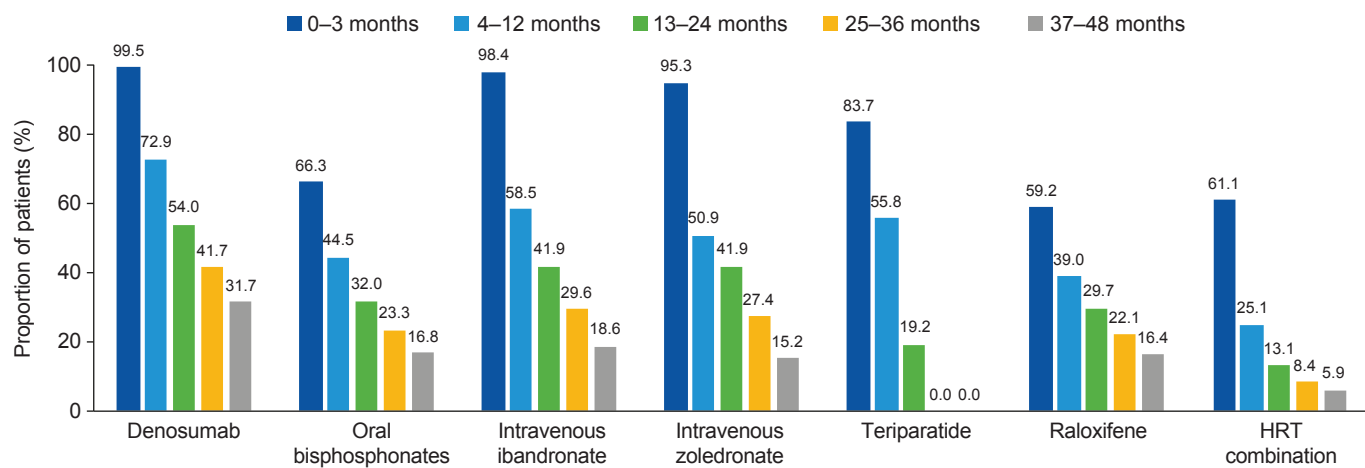

Supplement: Supplementary file 3 — Supplementary file3 (PDF 409 kb) [file 11657_2022_1156_MOESM3_ESM.pdf]

# All fractures

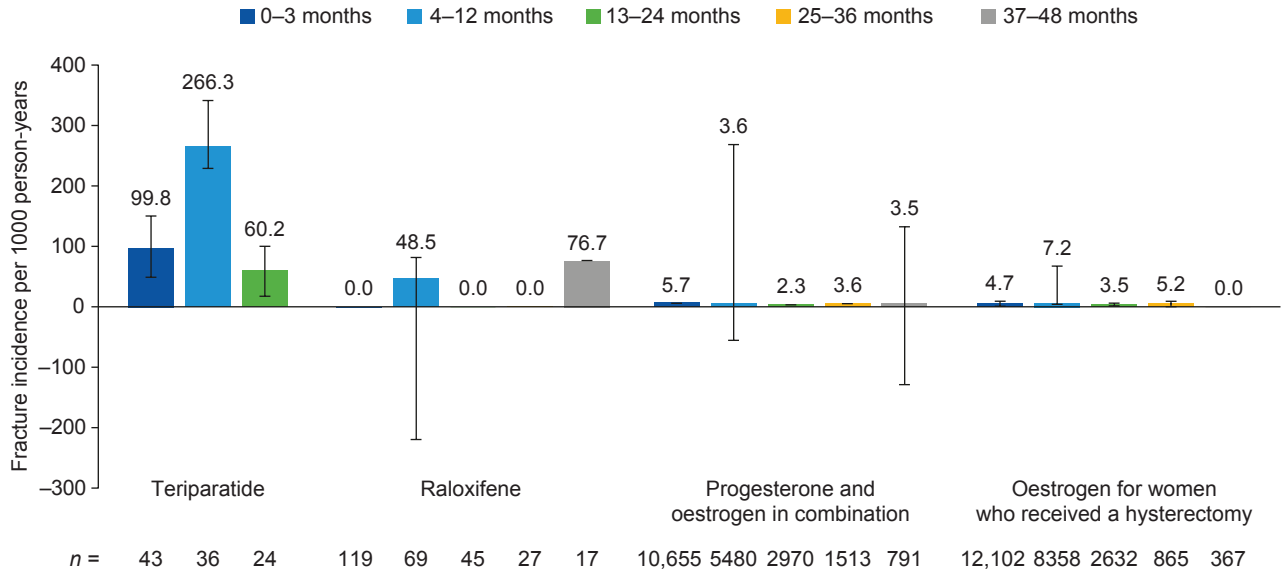

Supplement: Supplementary file 4 — Supplementary file4 (PDF 439 kb) [file 11657_2022_1156_MOESM4_ESM.pdf]

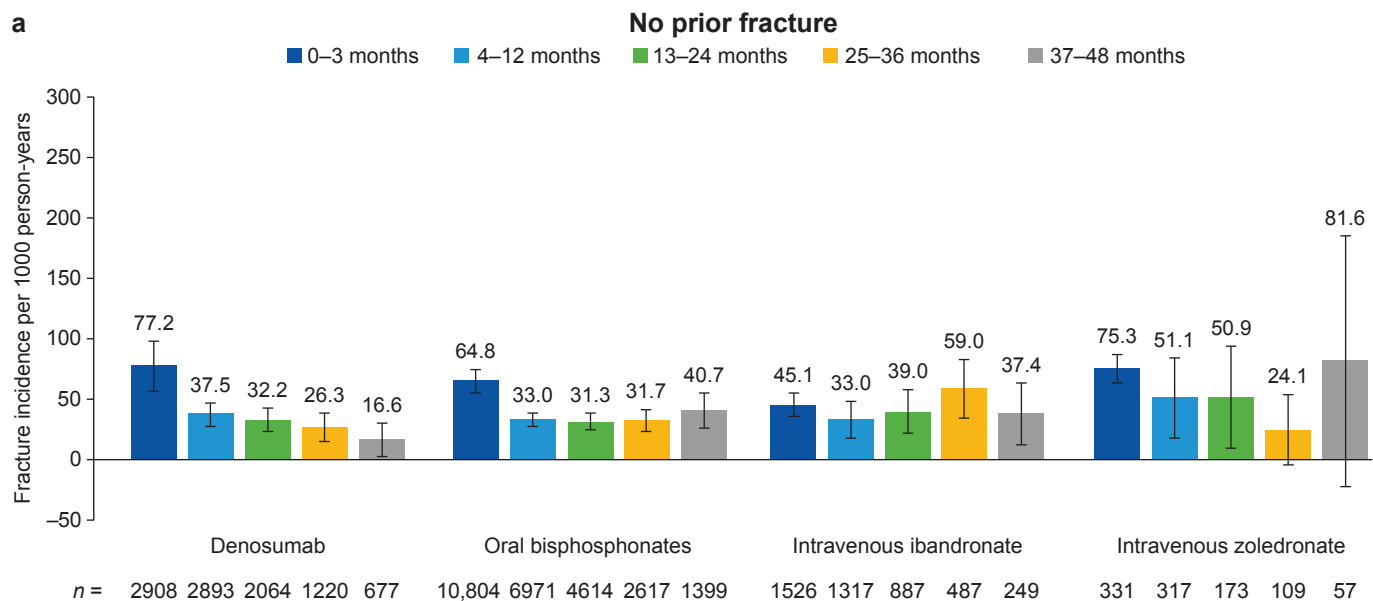

Supplement: Supplementary file 5 — Supplementary file5 (PDF 430 kb) [file 11657_2022_1156_MOESM5_ESM.pdf]

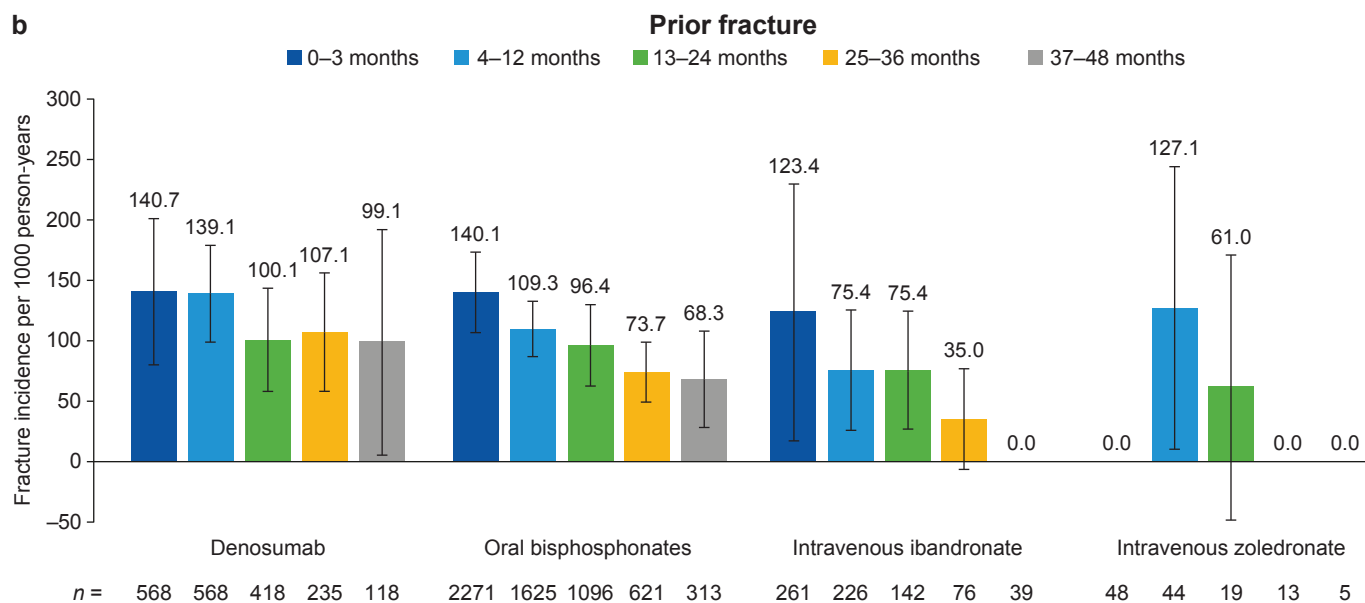

Supplement: Supplementary file 6 — Supplementary file6 (PDF 431 kb) [file 11657_2022_1156_MOESM6_ESM.pdf]

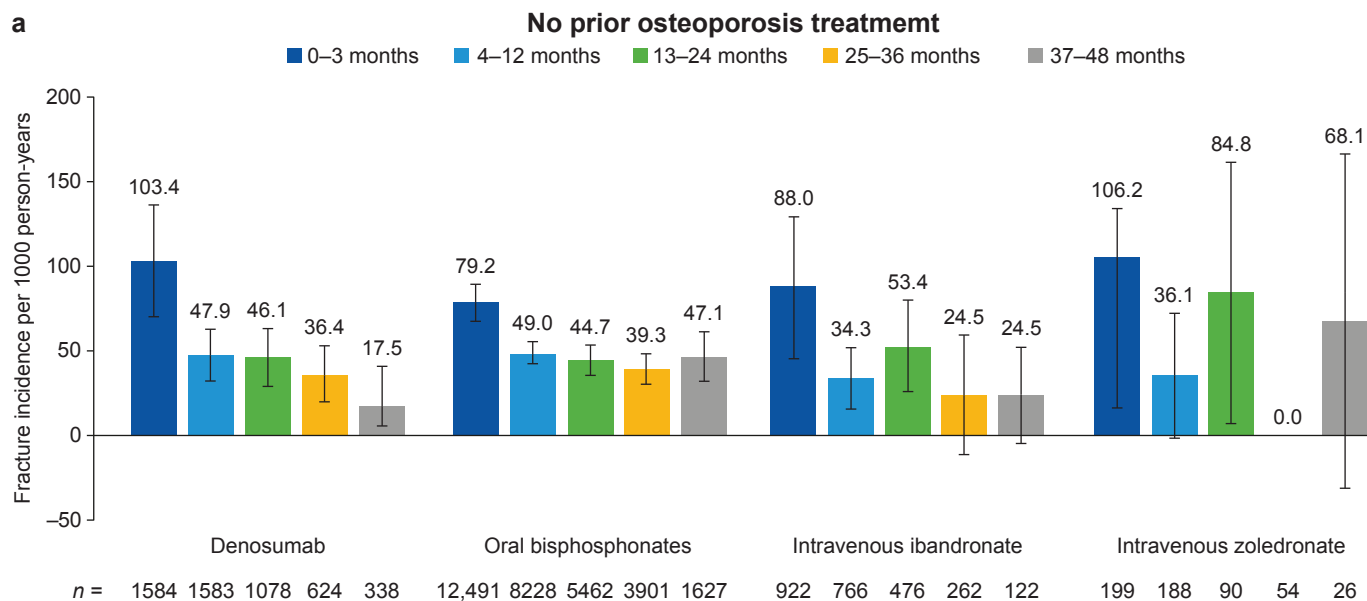

Supplement: Supplementary file 7 — Supplementary file7 (PDF 432 kb) [file 11657_2022_1156_MOESM7_ESM.pdf]

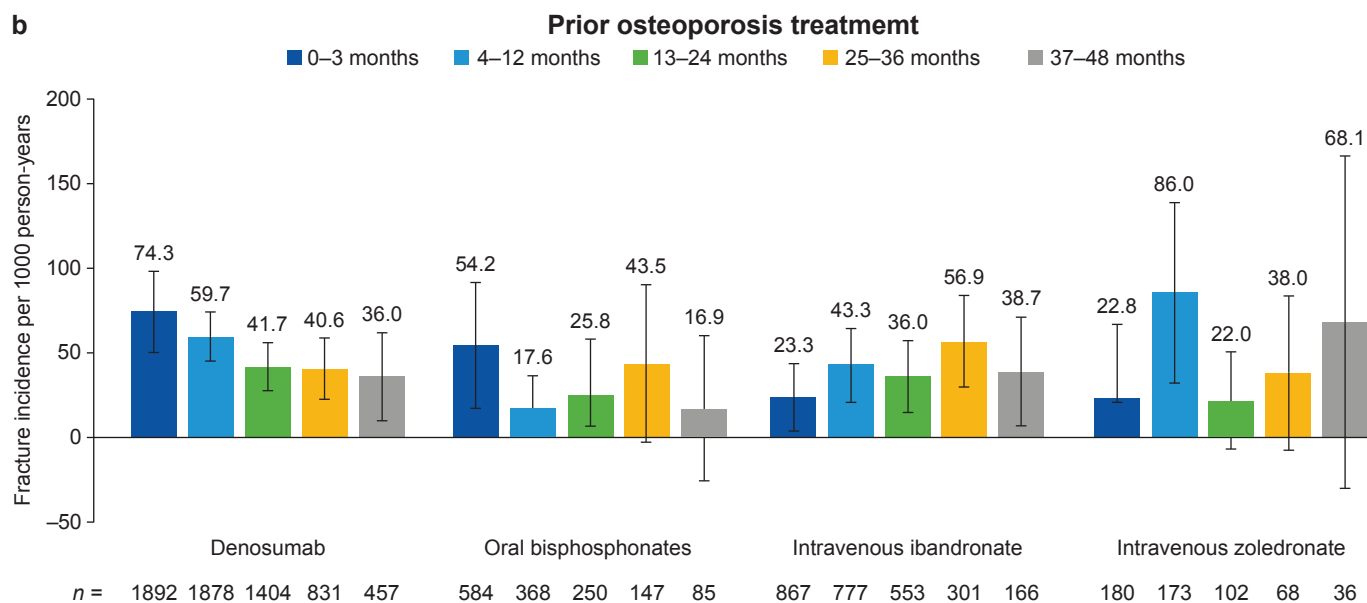

Supplement: Supplementary file 8 — Supplementary file8 (PDF 431 kb) [file 11657_2022_1156_MOESM8_ESM.pdf]
